# Virtual Reality-Based Sensory Stimulation for Pediatric Disorders of Consciousness: A Pilot Study

**DOI:** 10.3389/fped.2022.879422

**Published:** 2022-06-13

**Authors:** Piao Liang, Hong Xu, Sinan Li, Lei Ren, Xiaoke Zhao

**Affiliations:** ^1^Department of Rehabilitation, Children's Hospital of Nanjing Medical University, Nanjing, China; ^2^Department of Nursing, Children's Hospital of Nanjing Medical University, Nanjing, China

**Keywords:** virtual reality, sensory stimulation, pediatric, disorders of consciousness, pilot study

## Abstract

**Objective:**

The purpose of this study was to determine whether virtual reality-based sensory stimulation has the ability to improve the level of consciousness in pediatric disorders of consciousness compared with general rehabilitation.

**Methods:**

Thirty subjects were divided into a virtual reality (VR) group (*n* = 15) and a control group (*n* = 15). Subjects in the VR group received both general rehabilitation and exposure to VR videos; the control group received only general rehabilitation. The Glasgow Coma Scale (GCS), Coma Recovery Scale-Revised (CRS-R), and amplitude-integrated electroencephalogram (EEG) (aEEG) were used to measure the clinical behavioral response and neuroelectrophysiology before and after the treatment. The Glasgow Outcome Scale Extended Pediatric Revised (GOS-E Peds) was used to measure the social and personal functional ability after 3 months.

**Results:**

After 2 weeks of treatment, the CRS-R and GCS improved in both groups. However, the VR group had better results than the control group in the CRS-R (*p* = 0.003) and GCS (*p* = 0.045). There were no significant differences on aEEG in the two groups after treatment. According to the GOS-E Peds, the improvement of social and personal functional ability had no significant differences in the two groups. Additionally, there were no obvious adverse reactions in the two group during the treatment.

**Conclusions:**

This pilot study indicates potential benefit from the addition of VR to standard rehabilitation in pediatric disorders of consciousness. To further explore the efficacy of VR, a large-sample randomized controlled trial is warranted.

## Introduction

With the rapid development of emergency and critical care medicine and the continuous improvement of medical technology, the survival rate of brain injury due to various causes has greatly improved. However, many patients still have varying degrees of disorders of consciousness (DOC), such as coma, vegetative state (VS), and minimally conscious state (MCS). One cause of DOC in children is craniocerebral trauma as a result of events such as traffic accidents and falls. Non-traumatic causes include viral encephalitis and hypoxic ischemic encephalopathy. Children with DOC not only bring difficulties to nursing but are also prone to complications such as lung infection, urinary tract infection, joint contracture, skin acne, and malnutrition, which place heavy psychological and economic burdens on families of children who have had lengthy hospitalizations. Therefore, the treatment of pediatric DOC is one of the issues that intensive care unit and rehabilitation facility pay close attention to.

Some studies have reported the use of dopaminergic agonists (1, 2), γ-aminobutyric acid receptor agonists, and neuroregulatory techniques (3, 4) as methods for the treatment of DOC. However, there is still a lack of systematic research and sufficient evidence-based medical evidence to prove efficacy. At present, there is no unified treatment strategy for pediatric DOC. In terms of pharmacological treatment: zolpidem (5) and amantadine (6) have been reported to have the potential to improve the level of consciousness in children with consciousness disorders. Non-pharmacological treatments including median nerve stimulation (7), transcranial direct current stimulation (tDCS) (8), multi-sensory stimulation (9) were also used for children with DOC. However, due to the small sample sizes and single center study, there is insufficient evidence to demonstrate the safety and effectiveness of pharmacological and non-pharmacological treatments. Patients with DOC have disorders in self-perceptions and in perceiving or managing their surrounding environment. Scholars believe that creating a rich perceptual environment in the early stages of recovery can avoid delays in nerve repair caused by sensory deprivation (10). Rose et al. (11) also believed that environmental enrichment has a positive impact on the plasticity and functional recovery of the damaged brain, and suggested that virtual reality (VR) has great potential for creating a rich environment.

A VR system, which is defined as an interactive system that includes computers and media peripherals, can be used to create an environment that is similar to the real world, providing both audio and video stimuli to users (12). VR has three characteristics: immersive, interactive, and imaginative. The use of VR for therapy, rehabilitation, and training is increasing. However, it has been reported that 7 to 20% of users experience simulator sickness (nausea, oculomotor, disorientation) (13) while using head-mounted displays (HMD). Simulator sickness may be influenced by technological differences within HMDs such as resolution and refresh rate, and VR content also plays a significant role.

The game elements of virtual reality can increase a child's motivation for participation and treatment compliance. VR has been reported to have the ability to improve cognitive function in children with autism and motor function in children with cerebral palsy, and to offer pain relief (14–16). However, there have been few reports of the use and efficacy of VR in improving the consciousness of children with DOC. The aim of this pilot study was to determine whether general rehabilitation combined with virtual reality-based sensory stimulation has the ability to improve the level of consciousness in pediatric DOC compared with general rehabilitation. Our hypothesis for the study was that general rehabilitation and VR can improve the level of consciousness in children with DOC.

## Materials and Methods

### Participants

**Figure 1:**
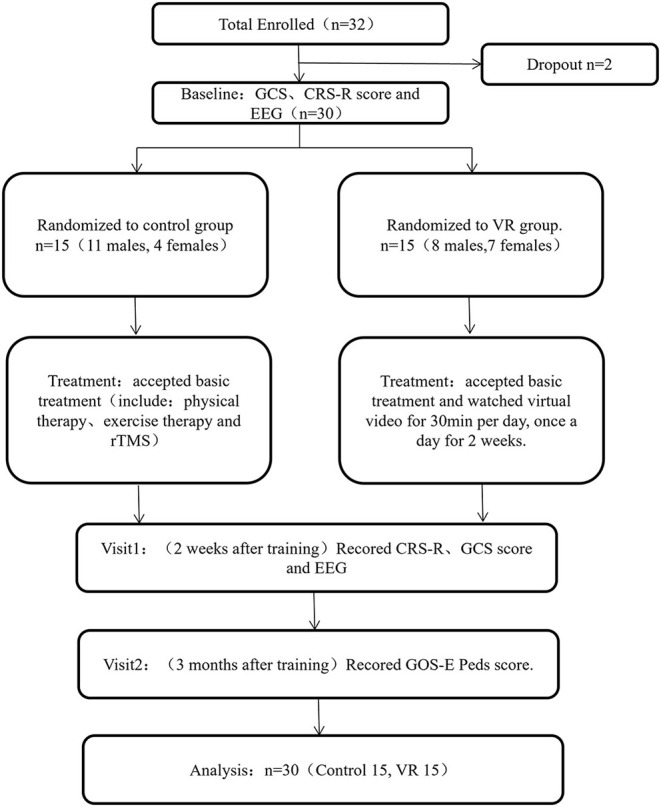
Diagram describing study flow. GCS, Glasgow Coma Scale; CRS-R, Coma Recovery Scale-Revised; EEG, electroencephalogram; TMS, transcranial magnetic stimulation; VR, virtual reality; GOS-E Peds, Glasgow Outcome Scale Extended Pediatric Revised.

Patients between 2 and 16 years of age hospitalized in the rehabilitation department, PICU and SICU of Nanjing Children's Hospital from January 2020 to December 2021 were recruited. The inclusion criteria were as follows: (a) according to the JFK Coma Recovery Scale-Revised (CRS-R) scores: Vegetative State (VS) (auditory ≤ 2 and visual ≤ 1 and motor ≤ 2 and verbal ≤ 2 and communicatio*n* = 0) or Minimally Conscious State (MCS) (auditory = 3–4 or visual = 2–5 or motor = 3–5 or verbal = 3, or communication = 1); and (b) stable vital signs. Patients were excluded if they had any of the following: (a) use of psychotropic drugs within 1 day; (b) suffered from neurological disorder or serious mental illness before brain injury; (c) severe visual and hearing impairment before or after brain injury; (d) poor treatment compliance; and (f) other severe complications during observation. A research flowchart is shown in Figure 1. Thirty-two patients who met the inclusion and exclusion criteria were included. However, two of them failed to complete the electroencephalogram (EEG) and were omitted. We used a computer program to generate 30 numbers that were randomly divided into two groups (VR and control). According to the sequence of enrollment, the first patient received a number, the second patient received another number, and so on. Patients in this study were randomly assigned to the VR group (*n* = 15; 8 male; 7 female) and the control group (*n* = 15; 11 male; 4 female).

Informed consent forms were signed by the legal guardians of all children participating in this study. This study was approved by the Ethics Committee of the Children's Hospital of Nanjing Medical University (batch number: 201912253-1). The registration number in the Chinese Clinical Trial Registry is ChiCTR2000034517.

The demographic and clinical characteristics of the participants are listed in Table 1. No significant differences were observed at baseline between the control and VR groups.

**Table 1:** Demographic and clinical characteristics at baseline.

**Characteristic**	**VR** **(*n =* 15)**	**Control** **(*n =* 15)**	***p-*value**
Age—yr, median (IQR)	6.1 (3.6–11.1)	4.8 (3.8–9.1)	0.885
Sex (*n*; %)			0.256
Male	8 (53.3)	11 (73.3)	
Female	7 (46.7)	4 (26.7)	
Duration of disease (days), median (IQR)	7 (5–10)	8 (4–16)	0.803
Etiology (*n*; %)			0.910
Traffic accidents injury	6 (40.0)	7 (46.6)	
Fall injury	4 (26.7)	4 (26.7)	
Viral encephalitis	5 (33.3)	4 (26.7)	

*Plus-minus values are means ± SD. There were no significant differences between the study groups (P > 0.05)*.

### VR Equipment

**Figure 2:**
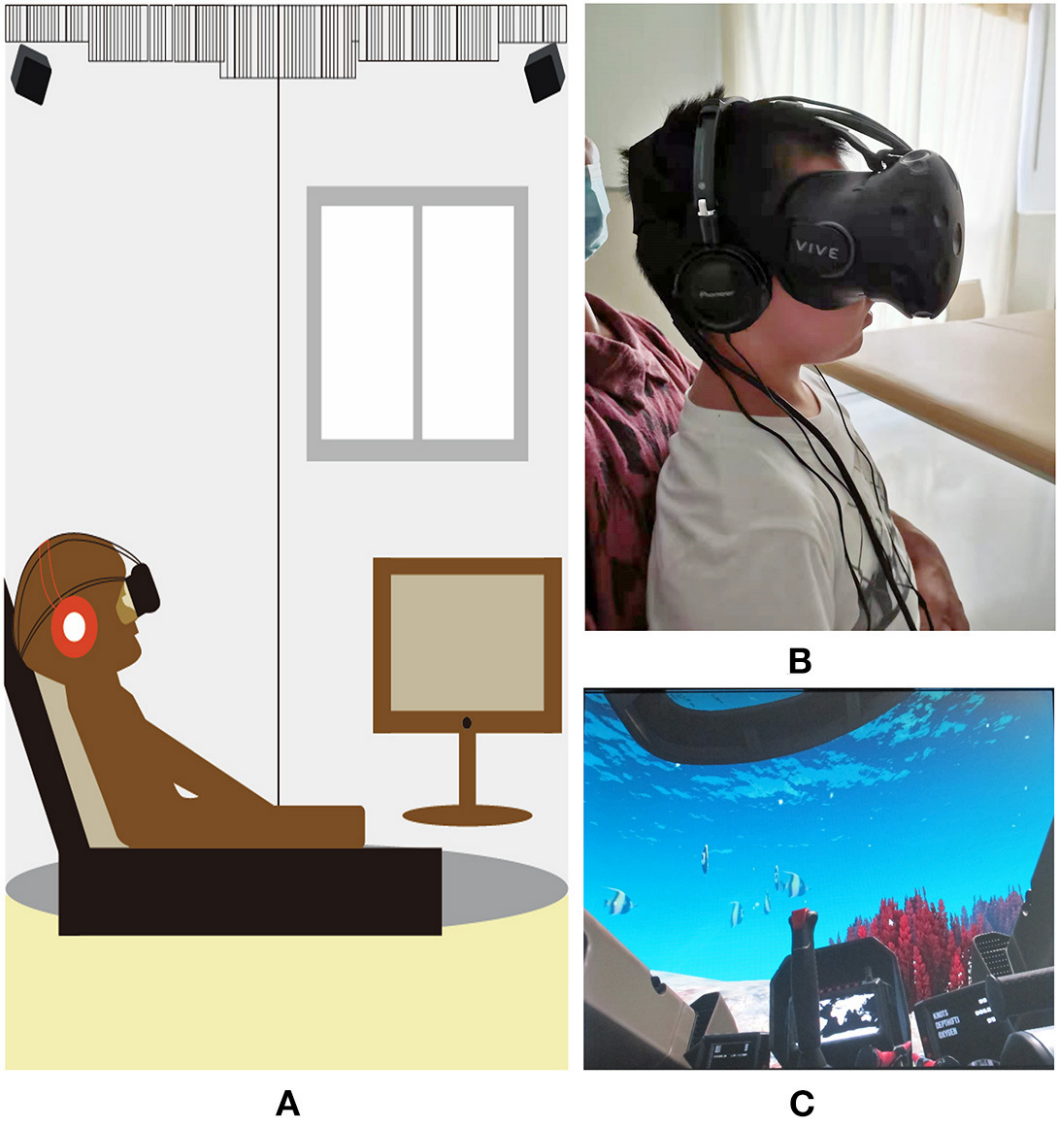
Virtual reality headset and pictures. **(A)** The virtual reality system used in the study. The system includes head-mounted equipment, control handles, infrared locators, computer; **(B)** The patient is watching virtual reality video; **(C)** A picture of “Submarine VR”.

The HTC Vive (Figure 2A) was used for this study, which includes head-mounted displays, handheld controllers, infrared locators, stereo equipment, and desktop computers. For safety purposes, all equipment was cleaned with alcohol-based sanitary wipes between patients. The VR intervention was implemented by trained and supervised research staff. VR videos included the following categories: personalized videos, panoramic videos (viewing videos and game videos), all of which provided visual, auditory, and vestibular stimuli. Personalized videos were provided by the family and panoramic videos were retrieved from the web. For patients in the PICU and SICU, study procedures were performed in the patients' ICU rooms, while those in the rehabilitation department performed in a separate room (Figures 2B,C).

### Intervention

All participants received general therapy including physical therapy, exercise therapy, and repetitive transcranial magnetic stimulation. In addition to general therapy, the VR group watched VR videos for 30 min per day for 2 weeks. Patients with continuous eye closure were subjected to pain stimulation (applyed pressure to face, neck, hand and foot muscles) for 1 min before therapy. To customize the stimulus to make sense to the individual, we asked the child's family and friends about their interests and habits prior to the DOC onset before the experiment began. All patients in the VR group watched personalized videos (for 15 min) first and then panoramic videos (for 15 min).

In the first stage, patients in the VR group watched personalized videos for 15 min, mainly providing visual and auditory stimuli. At the beginning of the personalized videos, children's parents called their names repeatedly and described interesting stories in a gentle tone. Parents also showed their daily life in the video, with activities such as cooking, attending parties, or visiting with friends. The personalized videos included videos recorded before the DOC. This part of the videos contained children's pictures, voices, and impressive events, which helped to reproduce scenes and were conducive to refresh their memories.

In the second stage, patients in the VR group watched panoramic videos for 15 min, which provided visual, auditory, and vestibular stimuli.

“Live in Your Music,” which is a music visualization game, allows patients to choose favorite songs and themes (pink butterflies, block driving, camping in the wild). When the patients put on a head mounted display, they can see a music environment with the theme they choose. Like the pink butterfly theme, the center of the field is a varied pattern of many pink butterflies. When the head is turned in any direction, the butterflies can be seen dancing, and the size and position of the butterflies change with the rhythm and sound level of the music.

“VR Roller Coaster at Global Wonders,” it includes multiple scenes (Rainforests, Lotus Pond, Zhangjiajie), and each scene contains different classical music. After wearing a head-mounted display, patients become a virtual character who rides on a roller coaster to experience different scenes. The patients can enjoy the scenery when the roller coaster is moving slowly, and experience the excitement when the roller coaster is speeding up.

“Submarine VR,” it is a game full of adventure under the sea. When the patients put on a head mounted display, patients are disguised as submarine drivers who are depicted as diving slowly into the deep sea and sailing on an endless ocean. When the submarine dives to the bottom, patients can see sunken ships, coral reefs, clownfish and mysterious sea creatures. And when the submarine nears the surface, patients can see rough waves, leaping dolphins and blue skies.

### Measurement

CRS-R and GCS scores were evaluated by a trained clinician before and after treatment, and the Glasgow Outcome Scale Extended Pediatric Revised (GOS-E Peds) was used to evaluate social and personal functional abilities after 3 months. And the EEG signals were monitored before and after treatment.

For children ages 2–5 the pediatric GCS was used, and for those older than 5 years the GCS was used (17). The scale includes eye opening, best motor response, and best verbal response and can quickly determine the initial level of consciousness (18).

The Coma Recovery Scale-Revised (CRS-R) consists of 23 items with six subscales addressing auditory, visual, motor, verbal, communication, and arousal functions (19, 20). The total score ranges from 0 to 23. The following scores indicate a diagnosis of VS: auditory ≤ 2, visual ≤ 1, motor ≤ 2, verbal ≤ 2, communicatio*n* = 0, and arousal ≤ 2. For the diagnosis of MCS, the scores are auditory, 3–4; visual, 2–5; motor, 3–5; verbal, 3; and communication, 1. The following scores indicate emergence from MCS (EMCS), the scores are: motor = 6 or communicatio*n* = 2 (21). This tool is the gold standard for the diagnosis of DOC, and is the best choice to distinguish VS/unresponsive wakefulness state (UWS) from MCS, as well as to observe subtle changes in consciousness (22).

In this study, the amplitude-integrated EEG (aEEG) were recorded as objective indices. According to Naqeeb et al.aEEG is divided into 3 grades: grade I, normal amplitude (aEEG upper edge >10 μV and aEEG lower edge >5 μV); grade II, mild abnormal amplitude (upper edge >10 μV and lower edge ≤ 5 μV, or upper edge ≤ 10 μV and lower edge >5 μV); and grade III, suppressed amplitude (upper edge <10 μV and lower edge <5 μV) (23).

We used the GOS-E Peds to measure social and personal functional ability before and after treatment, which provides an age-appropriate and effective neurologic measure for infants and children under 18 years of age (24). The list can be divided into three functional levels based on ratings: (1) no functional deficits (score = 1); (2) mild functional deficits (score = 2–3); and (3) significant functional deficits (score = 4–8) (25).

### Statistical Analysis

Data were analyzed using IBM SPSS Statistics 22.0. Results were summarized as frequencies and percentages for categorical variables, median and interquartile ranges for duration of disease, age, GCS, and CRS-R. Mann–Whitney U test was used to compare the change of aEEG and functional grade, GCS, and CRS-R. Chi-square test was used to compare the classification of consciousness levels before and after treatment. The *p* < 0.05 was reflected statistically significant. Bonferroni was applied to multiple comparison correction in the study.

## Results

### Participants

Thirty-two children were enrolled, but two failed to complete the EEG and were omitted. Thirty patients completed all treatment and evaluation follow-up tasks. As shown in Table 1, there was no significant difference in age, sex, etiology, duration of disease between the groups before treatment (*p* > 0.05). During the treatment, there were no obvious adverse reactions in the two groups. Only three children in the VR group showed mild irritability at the last stage of watching VR videos, which could be calmed after a short rest, while the other children did not show similar symptoms or other adverse reactions.

### CRS-R, GCS

As shown in Table 2, there was no significant difference in the score of CRS-R (*p* = 0.109) and GCS (*p* = 0.069) between the two groups before treatment. Patients who received general rehabilitation and exposure to VR videos got higher score in GCS (*p* = 0.045) and CRS-R (*p* = 0.003). And after the correction of Bonferroni, there was significant difference in CRS-R but no significant difference in GCS.

**Table 2:** The changes of CRS-R and GCS scores in both groups before and after treatment.

**Group**	**GCS**		**CRS-R**	
	**Pre**	**Post**	**Pre**	**Post**
VR (*n =* 15)	8 (7–10)	12 (11–15)	8 (3–8)	18 (15–23)
Control (*n =* 15)	6 (5–9)	11 (10–11)	3 (3–6)	9 (7–18)
*Z*	1.821	2.008	1.604	2.939
*p-*value	0.069	0.045	0.109	0.003

*The p-value refers to comparisons between VR group and Control group*.

### Consciousness

The classification of consciousness levels pre and post treatment can be seen in Table 3, patients with VS accounted for 33.3% and MCS accounted for 66.7% in the VR group while patients with VS accounted for 86.7% and MCS accounted for 13.3% in the control group before treatment. After treatment, patients with VS accounted for 0.0%, MCS accounted for 33.3%, EMCS accounted for 66.7% in the VR group while patients with VS accounted for 40.0%, MCS accounted for 33.3%, EMCS accounted for 26.7% in the control group. There were more patients classified as EMCS in the VR group compared to the control group. In the VR group, four patients from VS to MCS, one patient from VS to EMCS, one patient from MCS to MCS, and nine patients from MCS to EMCS. In the control group, six patients from VS to VS, four patients from VS to MCS, three patients from VS to EMCS, one patient from MCS to MCS, and one patient from MCS to EMCS. After the correction of *p-*values by Bonferroni, there was no significant difference before and after treatment in two groups.

**Table 3:** The classification of consciousness levels in two groups before and after treatment (n; %).

	**Pre**			**Post**		
	**VS**	**MCS**	**EMCS**	**VS**	**MCS**	**EMCS**
VR (*n =* 15)	5 (33.3)	10 (66.7)	0 (0.0)	0 (0.0)	5 (33.3)	10 (66.7)
Control (*n =* 15)	13 (86.7)	2 (13.3)	0 (0.0)	6 (40.0)	5 (33.3)	4 (26.7)
χ2			8.889			8.571
*p-*value			0.008			0.015

*The p-value refers to comparisons between groups. After the correction of p-values by Bonferroni, there was no significant difference before and after treatment in two groups*.

### aEEG

Both groups had the same ratio prior to intervention, as shown in Table 4, the proportion of normal aEEG (Grade I) was 60%, mild abnormal aEEG (Grade II) was 40%, and no patient was divided into suppressed amplitude (Grade III). After intervention, there were more people returning to normal aEEG (Grade I) as it accounted for 86.7% in the VR group and only 66.7% in the control group. And 13.3% patients stayed at mild abnormal aEEG (Grade II) in the VR group while 43.3% in the control group. However, there was no significant difference in the grade of aEEG in two groups after treatment (*p* = 0.203).

**Table 4:** The grade of amplitude integrated electroencephalogram (aEEG) in two groups before and after treatment (*n*; %).

**Group**	**Pre**		**Post**	
	**Grade I**	**Grade II**	**Grade I**	**Grade II**
VR (*n =* 15)	9 (60)	6 (40)	13 (86.7)	2 (13.3)
Control (*n =* 15)	9 (60)	6 (40)	10 (66.7)	5 (43.3)
*Z*		0.000		1.273
*p-*value		1.000		0.203

*The p-value refers to comparisons between groups*.

### The Functional Grade

As shown in Table 5, all patients had significant functional deficits before treatment, and there has no significant difference between groups (*p* = 1.000). Three months after the end of treatment, the proportion of patients with no functional defects in the VR group was higher than that in the control group. Moderate functional defects in the two groups were the same, while the number in the VR group with severe functional defects was lower than that in the control group. However, there has no significant difference between groups (*p* = 0.073).

**Table 5:** The functional grade in two groups during 3 months' follow-up (*n*; %).

**Group**	**Pre**			**Post**		
	**No functional deficits**	**Mild functional deficits**	**Significant functional deficits**	**No functional deficits**	**Mild functional deficits**	**Significant functional deficits**
VR (*n =* 15)	0 (0.0)	0 (0.0)	15 (100)	5 (33.3)	5 (33.3)	5 (33.3)
Control (*n =* 15)	0 (0.0)	0 (0.0)	15 (100)	1 (6.67)	5 (33.3)	9 (60.0)
*Z*			0.000			1.795
*P-*value			1.000			0.073

*The p-value refers to comparisons between VR group and Control group*.

## Discussion

To the best of our knowledge, this study is the first to use immersive VR to provide sensory stimulation for children with DOC in an attempt to improve the level of consciousness. The VR program involves visual, auditory, and vestibular stimuli to form multi-sensory stimuli, as well as a combination of music and memory therapy. We found that VR combined with general rehabilitation significantly increased the CRS-R score [auditory function from 1 (0–2) to 3 (2–4), visual function from 2 (0–2) to 4 (3–5), motor function from 2 (2, 3) to 5 (5, 6), verbal function from 1 (0–1) to 2 (1–3), communication from 0 (0–0) to 1 (1, 2), and arousal from 1 (1) to 3 (3)], among which, the auditory, verbal, visual, and arousal scores were significantly increased. Although there were differences in the classification of consciousness between the groups before treatment, all patients with VS in the VR group improved to MCS or EMCS, while 40% (6 patients) in the control group remained in VS. Salmani et al. (26) pointed out that early family-centered affective stimulation was more effective than single sensory stimulation in improving the level of consciousness and increasing CRS-R scores and improve the level of consciousness. Furthermore, familiar auditory stimuli were able to increase neural responsivity to vocal stimuli in language regions (27). Additionally, Heine et al. (28), suggest that preferred music exposure might have effects on patients auditory network (implied in rhythm and music perception) and cerebral regions linked to autobiographical memory. These previously reported results suggest that the improvement of consciousness level by sensory and music stimulation may mainly affect the auditory area of the brain. Our results suggest that this VR program may improve the level of consciousness, and auditory function in CRS-R, which is consistent with the results of previous studies.

Our result showed that there was no significant improvement in aEEG between groups after therapy. aEEG is the average value of the upper and lower boundaries of EEG amplitude, which directly reflects the severity of brain injury. A previous study has shown that VR training significantly improved both clinical and neurophysiological outcomes in patients with DOC, and the EEG plus VR approach used for patients with DOC could be promising to define the most appropriate stimulation protocol (29). Our results also suggest that VR training may improve aEEG in patients with DOC. In the VR group, more patients recovered from mild abnormal amplitude (Grade II) to abnormal amplitude (Grade I) than in the control group; however, there was no significant difference between groups, probably due to the small sample size and short treatment time. Additionally, there was no significant difference in the classification of function between groups after treatment. A prior study pointed out that GOS-E Peds showed a strong correlation with the GOS at 3 and 6 months, indicating that it would be more appropriate to assess function at 3 and 6 months after brain injury. In our study, drug exposures were similar between the two groups, which reduces the contribution of psychotropic medications to explain differences between groups. We plan to conduct a study with a large sample size and conduct longer follow-up time to clarify the effect of VR on aEEG and the functional recovery of children with DOC.

The traditional sensory stimuli for patients with DOC include visual, auditory, tactile, and olfactory, with stimuli such as relatives talking, providing familiar music, and offering views of simple pictures (27, 30). The traditional approach lacks an effective system for multisensory input. Some researchers suggested that richness and complexity of environmental stimuli play a key role in evoking active behaviors. The researchers used theatrical and artistic techniques to create an immersive environment for patients with DOC, and the results showed that familiar objects in an augmented environment elicited a greater range of behavioral responses (31). However, their study was a preliminary study with a small sample size and the authors failed to draw a scientific conclusion.

Many studies have reported that VR is an effective tool for providing multisensory stimulation. Yang et al. proposed a theoretical framework for a coma sensory stimulation program (CSP) system based on virtual reality. The theoretical system uses 3D modeling to create a virtual environment equipped with head-mounted displays, 3D trackers, stereo systems, olfactory stimulation devices, wearable tactile displays, and computers, which can provide strong visual, auditory, tactile, and olfactory stimulation (32). However, CSP is not yet used in clinical practice and the curative effect is unknown. In addition, Gerber et al. performed a study in which they equipped healthy adults with VR devices in the intensive care unit. The results showed that users relaxed under VR stimulation, and had no signs of fatigue during visual exploration. The authors suggested that visual and auditory stimulation based on VR is safe and has great potential in reducing psychological burden and restoring cognition and attention of critically ill patients (33). A recent case study reported a 17-year-old patient with DOC who received cognitive rehabilitation training with semi-immersive VR, and the results showed that both clinical and neurophysiological parameters were significantly improved (29).

In this study, we used VR to create immersive environments to produce multimodal sensory stimuli. For the panoramic video, the programs “Live in Your Music” and “Submarine VR” provided patients with visual, auditory, and vestibular stimuli; the personalized videos also provided patients with visual, auditory, and emotional stimuli. Environmental (sensory) deprivation has been reported to adversely affect synaptic remodeling (34). Previous studies have shown that familiar sensory and musical stimuli create a rich environment that improves neurobehavioral responses in patients with consciousness disorders (21–23). In addition, many researchers have suggested that patients in a coma or VS receiving intensive, repetitive multisensory stimulation will reach a higher threshold of reticular neurons, which is beneficial for an increase in cortical activity (35) and which activates the important “pathway” of the central loop related to awakening (36, 37). Our results showed that multisensory stimulation using VR significantly improved behavioral responses and neuroelectrophysiology in children with DOC and may also improve prognosis, which is consistent with the findings of previous studies.

Unfortunately, we did not conduct a questionnaire survey on virtual sickness (side/adverse effects) because the children in this study lacked accurate communication and poor coordination. Brooks et al. (38) stated that VR disease can manifest as disorientation, vertigo, nausea, vomiting, etc. In this study, we did not observe vomiting in our clinical observations. However, three children showed mild irritability in the later stages of watching the video, showing head and limb wriggling, and closing their eyes, which disappeared after a short rest, while the other children did not show similar symptoms or other adverse reactions. We thought the agitation could be caused by visual fatigue, vertigo, nausea or even improvement of consciousness.

There are some limitations to this study: Firstly, this was a pilot study and the sample size was small; therefore, any inferences based on the results should be made with caution. Secondly, despite attempts to limit the use of potentially psychotropic drugs for patients hospitalized in PICU or SICU, such drugs were used frequently. Exposure to psychotropic drugs may affect the therapeutic effect of VR or general rehabilitation. To reduce the acute impact of medications, no psychotropic medications were administered in the 24 h preceding treatment. However, in order to eliminate the drugs interference, future studies will include larger sample size and accounting for drug administration. Thirdly, we compared general rehabilitation and virtual reality combined with general rehabilitation, so we could not determine the degree to which the benefits of virtual reality are independent of or synergistic with such general rehabilitation. In the future, we need to take large sample study that compare only general rehabilitation and virtual reality to determine which one gives the main benefit in the further. Finally, this study used several scales mainly for clinical evaluation, and reactivity EEG (EEG-R) and aEEG were also used to objectively evaluate the changes of behavioral responses. However, the statistical comparison of EEG-R was not performed due to the lack of data before treatment. In addition to aEEG and EEG-R, more objective indicators should be taken to evaluate the curative effect such as body surface evoked potentials, brainstem auditory evoked potentials, event-related potentials, transcranial magnetic stimulation combined with EEG, and electromyography in the future studies.

## Conclusion

This pilot study indicates potential benefit from the addition of VR to standard rehabilitation in pediatric disorders of consciousness. Through this study, we have provided preliminary evidence to support that VR can improve the level of consciousness in pediatric DOC. The exact efficacy of VR is worthy of future randomized controlled trial studies with large sample sizes.

## Data Availability Statement

The raw data supporting the conclusions of this article will be made available by the authors, without undue reservation.

## Ethics Statement

This study was approved by the Ethics Committee of the Children's Hospital of Nanjing Medical University (batch number: 201912253-1). The registration number in the Chinese Clinical Trial Registry is ChiCTR2000034517. Written informed consent to participate in this study was provided by the participants' legal guardian/next of kin. Written informed consent was obtained from the individual(s), and minor(s)' legal guardian/next of kin, for the publication of any potentially identifiable images or data included in this article.

## Author Contributions

XZ and PL conceptualized the idea and designed experiment. PL, SL, and LR contributed to clinical data collection and assessment. PL and HX contributed to statistical analysis. PL wrote the manuscript. XZ approved the final manuscript. All authors read and approved the final manuscript.

## Funding

This work was supported by National Natural Science Foundation of China (81501946), Jiangsu Women and Children Health Association Scientific Research Foundation (FYX202013), and Nanjing Medical Science and Technology Development Foundation (YKK18144).

## Conflict of Interest

The authors declare that the research was conducted in the absence of any commercial or financial relationships that could be construed as a potential conflict of interest.

## Publisher's Note

All claims expressed in this article are solely those of the authors and do not necessarily represent those of their affiliated organizations, or those of the publisher, the editors and the reviewers. Any product that may be evaluated in this article, or claim that may be made by its manufacturer, is not guaranteed or endorsed by the publisher.
